# Optimization of Pixel Size and Electrode Structure for Ge:Ga Terahertz Photoconductive Detectors

**DOI:** 10.3390/s22051916

**Published:** 2022-03-01

**Authors:** Yifei Wu, Zuoru Dong, Yulu Chen, Bingbing Wang, Liming Wang, Xiaowan Dai, Junming Zhang, Xiaodong Wang

**Affiliations:** 1 Shanghai Microwave Technology Research Institute, Shanghai 200063, China; yfwu773@163.com (Y.W.); zrdong2021@163.com (Z.D.); wbb0308201@163.com (B.W.); monserdxw@126.com (X.D.); 2 Wide Bandgap Semiconductor Technology Disciplines State Key Laboratory, Xidian University, Xi’an 710071, China; lmwang@xidian.edu.cn (L.W.); probe1998@163.com (J.Z.)

**Keywords:** Ge-based terahertz photoconductive detector, pixel size, electrode structure, spectral response

## Abstract

To investigate the effects of the pixel sizes and the electrode structures on the performance of Ge-based terahertz (THz) photoconductive detectors, vertical structure Ge:Ga detectors with different structure parameters were fabricated. The characteristics of the detectors were investigated at 4.2 K, including the spectral response, blackbody response (*R_bb_*), dark current density-voltage characters, and noise equivalent power (NEP). The detector with the pixel radius of 400 μm and the top electrode of the ring structure showed the best performance. The spectral response band of this detector was about 20–180 μm. The *R_bb_* of this detector reached as high as 0.92 A/W, and the NEP reached 5.4 × 10^−13^ W/$\sqrt{\mathrm{Hz}}$ at 0.5 V. Compared with the detector with a pixel radius of 1000 μm and the top electrode of the spot structure, the *R_bb_* increased nearly six times, and the NEP decreased nearly 12 times. This is due to the fact that the optimized parameters increased the equivalent electric field of the detector. This work provides a route for future research into large-scale array Ge-based THz detectors.

## 1. Introduction

Terahertz (THz) detection is useful for many practical applications, such as astronomical observations, nondestructive testing, and biomedical treatment [1,2,3]. The impurity band detector as a typical extrinsic photoconductive THz detector is widely studied for its high sensitivity, wide spectrum, and fast response, which realizes detection by constructing an impurity band in a semiconductor energy gap and causing electrons to transition between the impurity band and the conduction band or valence band [4,5,6]. The Ge-based impurity detector is a promising candidate, in that the available impurity band in Ge is shallow enough to just absorb THz radiation (e.g., P in Ge at 0.013 eV and Ga in Ge at 0.014 eV) [7,8,9,10]. It has been developed widely with a cutoff wavelength of nearly 200 μm, and it has already been used in astronomical observations such as the ASTRO-F project and the Spitzer Space Telescope [11,12,13]. However, although the Ge-based impurity detector has been developed for many years, there is still a large gap compared with other terahertz detectors in terms of array scale, performance, and maturity [14,15], due to many fabrication difficulties and problems. For example, the typical pixel sizes of the reported Ge-based THz detectors are almost over 1 mm [16,17], which are too large to be arrayed on a large scale and lead to a complex refrigeration and optical system [18]. Therefore, the ground-based popularization and application of Ge-based THz detectors are limited and not even reported. Moreover, the performance of the detectors, such as responsivity, also needs to be optimized.

The structural parameters of the detectors play a significant role in improving the performance of the THz detector, such as pixel size, shape, electrode structure, and so on [19,20,21,22]. Unfortunately, little attention has been paid to the relationship between the structural parameters and the performance of Ge-based THz detectors. In this research, several measurements were made on the performance of the vertical structure Ge-based THz conductive detector with different structural parameters. 

Herein, vertical Ge-based photoconductive THz detectors with different structural parameters were fabricated, where the absorbing layer (Ga-doped Ge layer) was epitaxial grown on a highly conductive Ge substrate by PECVD (Plasma Enhanced Chemical Vapor Deposition). The characteristics of the detectors were investigated at 4.2 K, including the spectral response, blackbody responsivity (*R_bb_*), dark current density-voltage characters, and noise equivalent power (NEP). The Ge:Ga detectors showed a significantly broad spectral response band of about 20–180 μm (i.e., 1.6–15 THz). The detector with a pixel radius of 400 μm and the top electrode of the ring structure showed the best performance. The *R_bb_* of this detector reached as high as 0.92 A/W, and the NEP reached 5.4 × 10^−13^ W/$\sqrt{\mathrm{Hz}}$ at 0.5 V. Compared with the detector with a pixel radius of 1000 μm and the top electrode of the spot structure, the *R_bb_* increased nearly six times, and the NEP decreased nearly 12 times. This is due to the fact that the optimized parameters increased the equivalent electric field of the detector. 

## 2. Design and Fabrication

**Figure 1:**
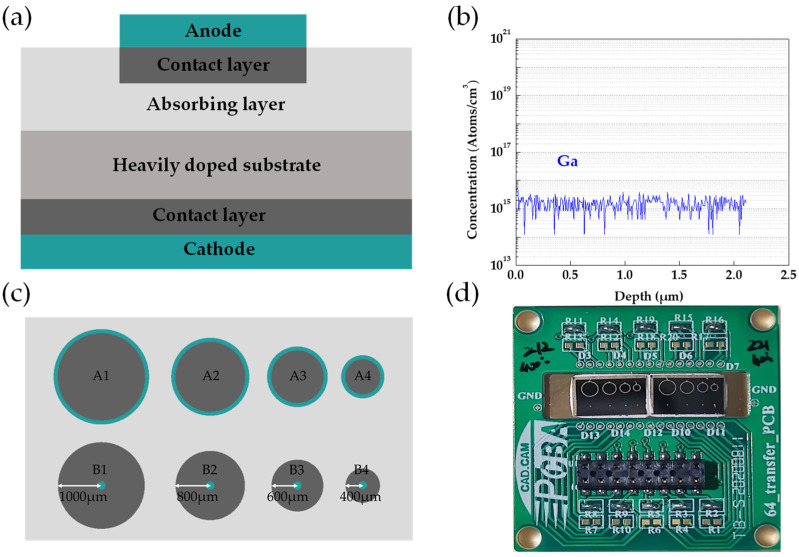
(**a**) Vertical structure of the Ge:Ga THz photoconductive detectors. (**b**) The SIMS result of the Ge epitaxial layer. (**c**) Top schematic of the detectors. (**d**) A photo of the detectors after packaging.

The vertical structure of the Ge:Ga THz photoconductive detector is shown in Figure 1a. In order to avoid the edge effect of electric field, the detector was designed to be circular. From top to bottom, each detector consisted of six parts, including an anode, an anode contact layer, a Ga-doped Ge absorption layer, a highly conductive Ge substrate, a cathode contact layer, and a cathode.

The absorption layer was fabricated by growing a 100 μm-thick Ge:Ga epitaxial layer on a highly conductive Ge substrate, with a Ga concentration of 1 × 10^15^ cm^3^. Figure 1b shows the secondary ion mass spectroscopy (SIMS) result of the Ge epitaxial layer. The doping concentration reached the expectation, and the doping Ga elements were uniformly distributed. 

After the absorption layer was prepared, a 20 nm SiO_2_ was sputter deposited on the absorption layer as a protective layer for ion implantation by PECVD. Both anode and cathode contact areas were fabricated by implanting B ions at a density of 2 × 10^14^ ions/cm^2^ at 40 keV. The wafer was then annealed at 400 °C for 2 min in an atmosphere of N_2_ by rapid thermal annealing, to recover the crystallinity and activate the B ions. Then the chip was soaked in 10% HF for 10 s to remove the SiO_2_ layer on the surface. The anode, consisting of 200 Å-thick Cr and 1800 Å-thick Au layers, was formed by thermal evaporation to optically separate the detector elements and to increase the conductance on the surface by electron beam evaporation. The 200 Å-thick Cr and 1800 Å-thick Au layers were also deposited on the backside of the Ge substrate as a cathode. Finally, the devices were annealed at 350 °C for 1 min in an atmosphere of N_2_ by rapid thermal annealing to form the metallic ohmic electrodes of the photoconductors.

As shown in Figure 1c, detectors with different structural parameters were fabricated to investigate the effects of the pixel sizes and the electrode structures on the performance of the Ge:Ga THz photoconductive detector. Considering the diffraction limitation and the whole device size, four different sizes of the detector with a radius of about 1000, 800, 600, and 400 μm were fabricated and labeled as 1, 2, 3, 4, respectively. To avoid the tip discharge of the electrode edge, the blocking of incident light, the electric field loss of asymmetrical electrode structure, and so on, the detector with the ring and the spot electrode structures were fabricated and labeled as A and B, respectively. The detector number and corresponding parameters are shown in Table 1.

The prepared detectors were packaged on the designed PCB board and connected with the external circuit through gold wires, as shown in Figure 1d. Then the packaged detectors were placed in the vacuum low-temperature Dewar system for performance testing at 4.2 K. 

## 3. Results and Discussion

**Figure 2:**
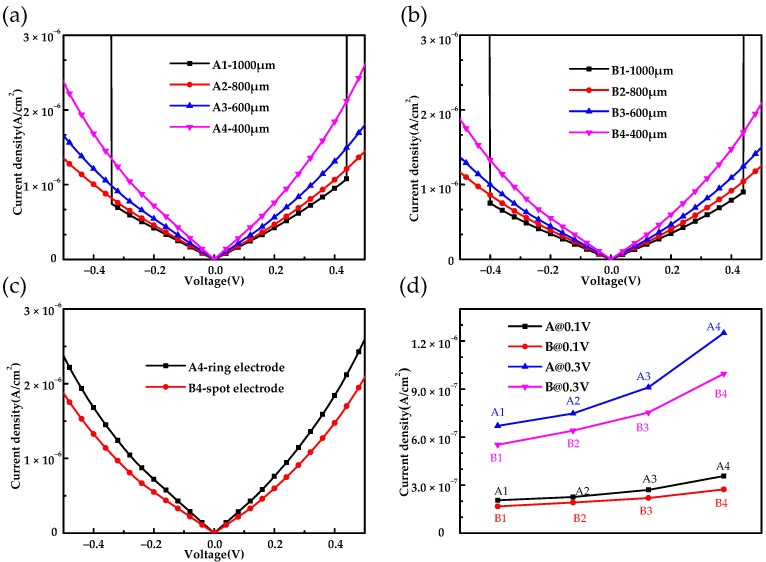
The dark current density-voltage characteristics of (**a**) A detectors and (**b**) B detectors. (**c**) Comparison of the dark current density-voltage of the A4 and B4 detectors. (**d**) The dark current density-voltage of the different detectors at 0.3 V and 0.5 V.

The dark current density-voltage characteristics of the Ge:Ga photoconductive detectors are shown in Figure 2. The current density is obtained by dividing the current by the pixel area. In Figure 2a,b, the dark current density-voltage curves under positive and negative electric fields are symmetrical, which is independent from the electrode structures (spot electrode or ring electrode). This indicates that the electrode and the device are well ohmic contacted. Meanwhile, the current density of A1 and B1 increased dramatically at 0.4 V due to the appearance of the avalanche breakdown effect. In addition, the current density of the detector increased as the pixel size decreased. This is because the ion implantation layer was thin and nonideal, and, thus, the electric field distribution was nonuniform, and there was electric field loss [23,24,25]. The electric field loss of large size detectors is greater than that of small detectors, so the equivalent electric field intensity of large detectors is smaller than that of small detectors. For the same reason, the equivalent electric field intensity of the spot electrode is smaller than that of the ring electrode. Therefore, the current density of the detector with a ring electrode was greater than that of the detector with a spot electrode, as shown in Figure 2c. Figure 2d shows the dark current density of the detectors with different structural parameters at 0.1 V and 0.3 V, and the phenomenon discussed above can be clearly seen from it. 

**Figure 3:**
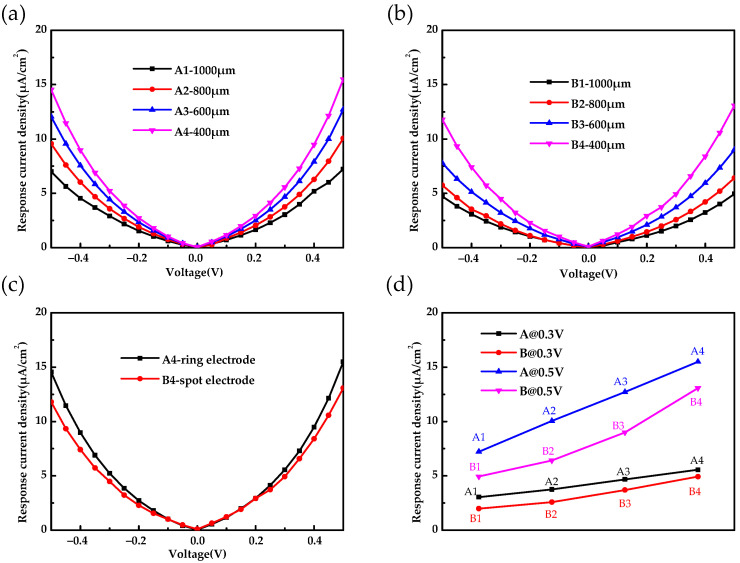
The blackbody response current density-voltage characteristics of (**a**) A detectors and (**b**) B detectors. (**c**) Comparison of the response current density of the A4 and B4 detectors. (**d**) The response current density of the different detectors at 0.3 V and 0.5 V.

The blackbody response current density-voltage characteristics of the Ge:Ga photoconductive detectors are shown in Figure 3. As shown in Figure 3a,b, the blackbody response current density-voltage curves under positive and negative electric fields were approximate symmetrical, and the current density of the detector increased as the pixel size decreased, as with the dark current density-voltage characteristic. As shown in Figure 3c, the current density of the detector with the ring electrode was greater than that of the detector with the spot electrode. Figure 3d shows the response current density of the detectors with different structural parameters at 0.3 V and 0.5 V, and the variation trend was the same as the dark current density. 

**Figure 4:**
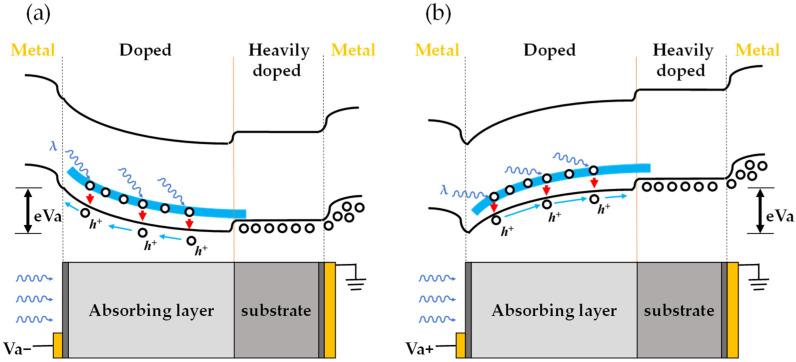
Schematic diagram of the energy band structure and detection mechanism of the Ge:Ga photoconductive detector under negative bias voltage (**a**) and positive bias voltage (**b**).

The schematic diagrams of the energy band structure and detection mechanism of the Ge:Ga photoconductive detector under negative and positive bias voltage are shown in Figure 4a,b, respectively. Under blackbody irradiation, electrons in the valence band were excited to the Ga-doped impurity band, thereby generating holes in the valence band. When a negative bias voltage was applied, the generated holes in the valence band were collected by the anode under the action of a negative electric field, thus forming a response current, as shown in Figure 4a. When a positive bias voltage was applied, the holes entered the highly conductive substrate under the positive electric field and then were collected by the cathode forming current, as shown in Figure 4b. The approximately symmetric blackbody response current density-voltage curves under positive and negative electric fields, shown in Figure 3, indicate that the doping impurity band of the highly conductive Ge substrate degenerated; that is, the doping concentration of the substrate was high enough that it had an extremely small resistance value and worked as a conductor, and the electrodes were well ohmic contacted. 

The *R_bb_* of the detector can be calculated by the formula [26,27,28]:

(1) $$R_{bb}=\frac{I_{PC}}{P_{B}}=\frac{I_{PC}}{\sigma \cdot (T_{2}^{4}-T_{1}^{4})\cdot A_{b}\cdot A_{d}/2\sqrt{2}\pi L^{2}}=\frac{i_{PC}}{\sigma \cdot (T_{2}^{4}-T_{1}^{4})\cdot A_{b}/2\sqrt{2}\pi L^{2}}$$

where *I_pc_* is the measured photocurrent, which is the blackbody response current, *P_B_* is the black-body radiation power received by the detector, *T*_2_ = 800 K is the blackbody temperature, *T*_1_ = 300 K is the background temperature, *σ* = 5.67 × 10^–12^ W/(cm^2^·K^4^) is the Stefan-Boltzmann constant, *A_b_* = 20 mm^2^ is set as the blackbody exit aperture area, *A_d_* is the effective photosensitive area, which is equal to the pixel size, *L* = 10 cm is the distance from the detector to the blackbody, and *i_pc_* is the blackbody response current density of the detector. Theoretically, detectors with different pixel radii should have the same responsivity, but the electric field loss induced by the actual process deviation will somehow affect the responsivity.

**Figure 5:**
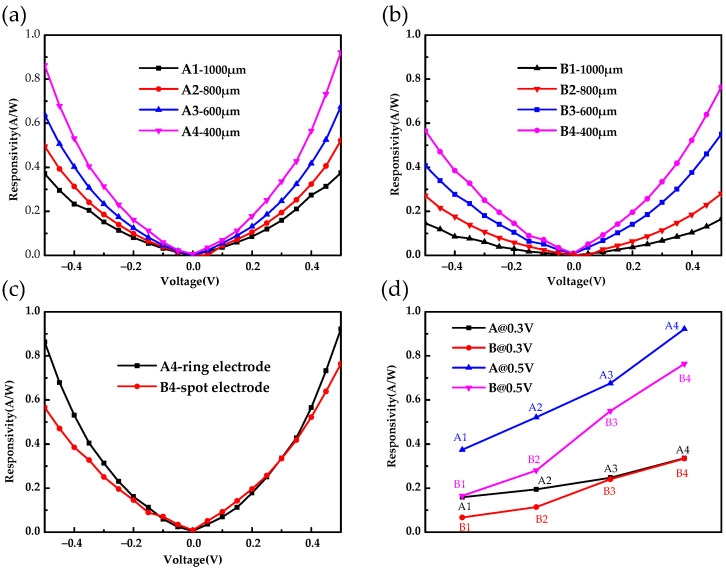
The blackbody responsivity of (**a**) A detectors and (**b**) B detectors. (**c**) Comparison of the blackbody responsivity of the A4 and B4 detectors. (**d**) Blackbody responsivity of the different detectors at 0.3 V and 0.5 V.

The *R_bb_* of the Ge photoconductive detectors with various pixel sizes and electrode structures are shown in Figure 5. Figure 5a,b shows that the *R_bb_* increases with the increase in bias voltage, and it increases with the decrease in detector size. The responsivity of the A4 detector with the pixel radius of 400 μm and the top electrode of the ring structure reached its maximum, that is, 0.92 A/W at 0.5 V and 4.2 K. Compared with the detector with the pixel radius of 1000 μm and the top electrode of the spot structure, the *R_bb_* increased nearly six times. Figure 5c shows the *R_bb_* of the A4 and B4 detectors at different voltages. For the same pixel size, the *R_bb_* of the detector with the ring electrode was larger than that of the detector with the spot electrode at the same voltage. This is due to the fact that the equivalent electric field intensity of the detector with the spot electrode is smaller than that of the detector with the ring electrode. Figure 5d intuitively shows the *R_bb_* of detectors with different structural parameters at 0.3 V and 0.5 V. 

**Figure 6:**
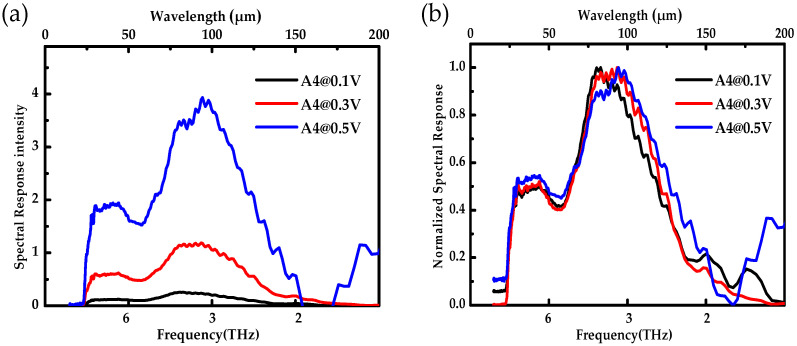
(**a**) The response spectra of the Ge:Ga THz photoconductive detectors. (**b**) The normalized response spectra of the Ge:Ga THz photoconductive detectors.

The spectral response intensity of the Ge-based photoconductive detector A4 at 0.1, 0.3, and 0.5 V are displayed in Figure 6a. The spectral response intensity of the detector increased exponentially as the electric field increased, which is consistent with the *R_bb_* of the detector.

The normalized response spectra are displayed in Figure 6b, which were obtained by dividing each response spectrum by the corresponding spectral peak value. The response peaks were located at 82, 88, and 94 μm, respectively. The peak wavelength, *λ_C_*, of the impurity band photoconductive detector can be calculated by the formula,

(2) $$\lambda_{C}=\frac{hc}{E_{A}}=\frac{1.24}{E_{A}}\;(\mu m)\;$$

where *h* is the Planck constant, *c* is the velocity of light, and *E_A_* (eV) is the activation energy of the impurity band. It can be calculated that the activation energy of the impurity band in Ge was about 0.013–0.015 eV, which corresponds to the activation energy of the Ga impurity band in Ge [29]. With the increasing bias, the response wavelength range of the detector was widened. This is because the higher bias produced more photo-generated current, resulting in the temperature rise of the detector, the lattice movement acceleration, and the impurity energy band broadening. As the temperature increases, the lattice movement becomes more intense, and the impurity energy band is widened.

In addition, a shoulder peak was observed between 30 μm and 40 μm, corresponding to the energy of 0.3–0.4 eV. After etching the electrode of the detector, it was found that the Au element of the electrode diffused into the absorption layer, and the energy of 0.3–0.4 eV corresponds to the activation energy of the Au impurity band in Ge [30,31,32]. Therefore, it can be inferred that the shoulder peak was caused by the electrons transition between the Au impurity band and the valence band of Ge, thereby broadening the detection band of the Ge-based THz photoconductive detector.

According to the NEP formula [33,34],

(3) $$\mathrm{NEP}=N_{DET}\frac{G}{R_{bb}}$$

where *R_bb_* is the blackbody responsivity, *N_DET_* is the detector noise, and *G* = 0.17 is the ratio of the peak response to the total response. Table 2 shows the *N_DET_* and NEP of each detector at 0.5 V and 4.2 K. It can be seen that the *N_DET_* and NEP of the detector decreased with the reduction in pixel size. In addition, for detectors with the same pixel size, the *N_DET_* of the different electrode detectors was almost the same, while the NEP of the ring electrode detector was smaller than that of the spot electrode detector, and the *R_bb_* of the ring electrode detector was larger than that of the spot electrode detector. The NEP of the A4 detector reached 5.4 × 10^−13^ W/$\sqrt{\mathrm{Hz}}$ at 0.5 V. Compared with the detector with the pixel radius of 1000 μm and the top electrode of the spot structure, the NEP decreased nearly 12 times. This indicates that the detector with the smallest pixel size and the top electrode of the ring structure showed the best performance. Based on the results in this work, a high-quality, small-pixel-size, and large-scale-array Ge-based photo-conductive detector will be fabricated in the future.

## 4. Conclusions

The vertical structural Ge THz photoconductive detectors were reported. The performance of detectors with various pixel sizes and electrode structures were investigated. The detector with the pixel radius of 400 μm and the top electrode of the ring structure showed a 20–180 μm spectral response band, 0.92 A/W responsivity, and the noise equivalent power of 5.4 × 10^−13^ W/$\sqrt{\mathrm{Hz}}$ at 0.5 V. Compared with the detector with the pixel radius of 1000 μm and the top electrode of the spot structure, the *R_bb_* increased nearly six times, and the NEP decreased nearly 12 times. This is due to the fact that the optimized parameters increased the equivalent electric field of the detector. It was verified that the optimization of the structural parameters plays a pivotal role in improving the sensitivity of THz photoconductive detector.

## Figures and Tables

**Table 1:** Detector number and the corresponding parameters.

Detector Number	Pixel Radius (μm)	Electrode Structure
A1	1000	Ring electrode
A2	800	
A3	600	
A4	400	
B1	1000	Spot electrode
B2	800	
B3	600	
B4	400	

**Table 2:** NEPs of the detectors.

Detector Number	$N_{\mathrm{DET}}\;(A/\sqrt{\mathrm{Hz}})$	$\mathrm{NEP}\;(W/\sqrt{\mathrm{Hz}})$
A1	6.5 × 10^−12^	2.9 × 10^−12^
A2	5.3 × 10^−12^	1.7 × 10^−12^
A3	4.4 × 10^−12^	1.1 × 10^−12^
A4	2.9 × 10^−12^	5.4 × 10^−13^
B1	6.2 × 10^−12^	6.3 × 10^−12^
B2	5.0 × 10^−12^	3.0 × 10^−12^
B3	4.1 × 10^−12^	1.3 × 10^−12^
B4	2.8 × 10^−12^	6.3 × 10^−13^
